# For debate: substituting placebo controls in long-term Alzheimer's prevention trials

**DOI:** 10.1186/alzrt68

**Published:** 2011-03-21

**Authors:** René Spiegel, Manfred Berres, André R Miserez, Andreas U Monsch

**Affiliations:** 1 University Hospital Department of Geriatrics, Memory Clinic, Schanzenstrasse 55, CH 4031 Basel, Switzerland; 2 University of Applied Sciences Koblenz, RheinAhrCampus Remagen, Südallee 2, D-53424 Remagen, Germany; 3 diagene Laboratories Inc., Kägenstrasse 17, CH 4153 Reinach, Switzerland

## Abstract

**Introduction:**

Novel compounds with potential to attenuate or stop the progression of Alzheimer's disease (AD) from its presymptomatic stage to dementia are being tested in man. The study design commonly used is the long-term randomized, placebo-controlled trial (RPCT), meaning that many patients will receive placebo for 18 months or longer. It is ethically problematic to expose presymptomatic AD patients, who by definition are at risk of developing dementia, to prolonged placebo treatment. As an alternative to long-term RPCTs we propose a novel clinical study design, termed the placebo group simulation approach (PGSA), using mathematical models to forecast outcomes of presymptomatic AD patients from their own baseline data. Forecasted outcomes are compared with outcomes observed on candidate drugs, thus replacing a concomitant placebo group.

**Methods:**

First models were constructed using mild cognitive impairment (MCI) data from the Alzheimer's Disease Neuroimaging Initiative (ADNI) database. One outcome is the Alzheimer Disease Assessment Scale - cognitive subscale (ADAScog) score after 24 months, predicted in a linear regression model; the other is the trajectory over 36 months of a composite neuropsychological test score (Neuro-Psychological Battery (NP-Batt)), using a mixed model. Demographics and clinical, biological and neuropsychological baseline values were tested as potential predictors in both models.

**Results:**

ADAScog scores after 24 months are predicted from gender, obesity, Functional Assessment Questionnaire (FAQ) and baseline scores of Mini-Mental State Examination, ADAScog and NP-Batt with an *R*^2 ^of 0.63 and a residual standard deviation of 0.67, allowing reasonably precise estimates of sample means. The model of the NP-Batt trajectory has random intercepts and slopes and fixed effects for body mass index, time, apolipoprotein E4, age, FAQ, baseline scores of ADAScog and NP-Batt, and four interaction terms. Estimates of the residual standard deviation range from 0.3 to 0.5 on a standard normal scale. If novel drug candidates are expected to diminish the negative slope of scores with time, a change of 0.04 per year could be detected in samples of 400 with a power of about 80%.

**Conclusions:**

First PGSA models derived from ADNI MCI data allow prediction of cognitive endpoints and trajectories that correspond well with real observed values. Corroboration of these models with data from other observational studies is ongoing. It is suggested that the PGSA may complement RPCT designs in forthcoming long-term drug studies with presymptomatic AD individuals.

## Introduction

A number of compounds with potential to attenuate the progression of Alzheimer's disease (AD) from a presymptomatic stage to dementia - that is, drugs intended for secondary prevention of dementia due to AD - are currently undergoing testing in man [1,2]. The study design routinely applied in advanced stages of clinical development (late phase 2, phase 3) of central nervous system active compounds is that of the randomized, placebo-controlled clinical trial (RPCT), a procedure implying that a high proportion of patients, up to 50% of the total sample, will receive inactive drug throughout. Given that meaningful study of experimental treatment intended for secondary prevention of dementia due to AD will take 18 months or more for each individual, it is problematic, from an ethical standpoint, to expose patients with mild cognitive impairment (MCI) and similar conditions, who by definition run a high risk of developing dementia, to prolonged exposure to placebo [3]. In addition, the external validity (representativity) of long-term RPCTs may be questioned, as many potential trial participants will decline inclusion in a study that intentionally exposes them to the risk of prolonged inactive treatment.

Although several groups of investigators have discussed more focused and/or more time-economical approaches to testing potential AD course-altering treatments [4-8], including variations of the conventional parallel-group clinical study design, little or no attention has been paid to the two fundamental problems of long-term RPCTs in high-risk individuals: the ethical issue of extended placebo exposure, and the problem of the trials' external validity. Here we propose a novel clinical strategy - the placebo group simulation approach (PGSA) - thought to be a viable alternative to long-term RPCTs and able to overcome a serious ethical and scientific dilemma of current clinical research in AD and similar conditions. Making use of anamnestic, biological, neuropsychological and other subject data routinely established at study baseline, the PGSA comprises mathematical modeling and forecasting of typical AD disease trajectories from its presymptomatic to symptomatic stages. Based on such forecasts, the endpoints and trajectories of patients undergoing experimental treatment intended for secondary prevention of dementia due to AD can be compared with their own modeled disease course; that is, with their predicted endpoints and trajectories had they not been treated. Based on these comparisons between observed and model-based outcomes, the efficacy of putative AD course-altering drugs can be delineated.

The present article describes the development of the predictive models, based on a large, multidimensional dataset collected from individuals characterized as MCI subjects - that is, presymptomatic individuals with a high risk to develop dementia within a few years. As will be noted, the PGSA differs from traditional historical control and observation studies with regard to three critical aspects. First, this approach uses mathematical modeling to identify and quantify those measures at baseline that allow one to forecast cognitive and/or other clinically relevant outcomes after a predetermined time period. Second, based on a variety of measures established at baseline, the PGSA provides a quantified prediction as to the expected time course of the outcomes selected by the investigators. Third, the predictive models of the PGSA are based on large sets of uniformly collected longitudinal observational data of properly defined patient samples. Furthermore, although the PGSA could be expanded to other areas of medicine where longitudinal data allowing computation of respective models are available, the focus of the present paper is on AD.

## Materials and methods

### Subjects and procedures

Data used in the preparation of this article were obtained from the Alzheimer's Disease Neuroimaging Initiative (ADNI) database [9]. The ADNI was launched in 2003 by the National Institute on Aging, the National Institute of Biomedical Imaging and Bioengineering, the Food and Drug Administration, private pharmaceutical companies and nonprofit organizations. The primary goal of ADNI has been to test whether serial magnetic resonance imaging, positron emission tomography, other biological markers, and the progression of mild cognitive impairment and early Alzheimer's disease. Determination of sensitive and specific markers of very early AD progression is intended to aid researchers and clinicians in developing new treatments and monitoring their effectiveness, as well as to lessen the time and cost of clinical trials.

The Principal Investigator of this initiative is Michael W Weiner, MD (VA Medical Center and University of California - San Francisco, CA, USA). The ADNI is the result of efforts of many co-investigators from a broad range of academic institutions and private corporations, and subjects have been recruited from over 50 sites across the United States and Canada. The initial goal of the ADNI was to recruit 800 adults, ages 55 to 90, to participate in the research - approximately 200 cognitively normal older individuals to be followed for 3 years, 400 people with MCI to be followed for 3 years and 200 people with early AD to be followed for 2 years. Up-to-date information is available online [9].

For the present analyses, we focus on the MCI subjects in the ADNI database. Participants were classified as MCI in the ADNI project when they had a Mini-Mental State Examination (MMSE) [10] score between 24 and 30, a memory complaint, a memory loss measured by education-adjusted scores on the Wechsler Memory Scale - Logical Memory II, a Clinical Dementia Rating [11] of 0.5, an absence of significant levels of impairment in other cognitive domains, essentially preserved activities of daily living, and an absence of dementia [12].

There was a total of 397 subjects with MCI at baseline (Table 1). These individuals were followed up at 6, 12, 18, 24 and 36 months. Data from 286 subjects with evaluations at baseline and at month 24 were available for an endpoint-related univariate analysis of outcomes after 2 years, and there were 375 subjects available with baseline and at least one postbaseline evaluation for a trajectory-related multivariate model (status October 2009). A total of 199 of the 397 subjects had undergone lumbar puncture at baseline, and 189 of these had at least one postbaseline cognitive testing. Note that a number of these MCI subjects had functional impairments at baseline (as indicated by elevated Functional Assessment Questionnaire (FAQ) scores) and that more than one-half of them took relevant medication for some time - specifically cholinesterase inhibitors or memantine, or a combination of these drugs.

**Table 1:** Demographic and baseline data for the mild cognitive impairment group

Characteristic	Value
	(*n *= 397)
Age (years)	74.2 (7.4)
Women	141 (36%)
Apolipoprotein E4	
0 E4 alleles	185 (47%)
1 E4 allele	165 (42%)
2 E4 alleles	47 (12%)
Education (years)	15. 7 (3.0)
Body mass index (kg/m^2^)	26.1 (4.0)
Hachinski modified (0 to 12)	1 (0 to 4)
FAQ (0 (normal) to 50)	2 (0 to 21)
MMSE (0 to 30 (best))	27.0 (1.8)
ADAScog, modified (0 (best) to 85)	18.6 (6.3)
NP-Batt (*z *score)	-1.02 (0.66)
Medication	240 (60%)
	(*n *= 199)
β-amyloid_1-42_	146.9 (48.3 to 298.8)
Total tau protein	85.6 (28.5 to 463.2)
β-amyloid_1-42_/total tau protein	1.564 (0.233 to 7.61)

Data presented as mean (standard deviation), *n *(%) or median (minimum to maximum). ADAScog, Alzheimer Disease Assessment Scale - cognitive subscale; FAQ, Functional Assessment Questionnaire; MMSE, Mini-Mental State Examination; NP-Batt, Neuro-Psychological Battery.

### Baseline data and neuropsychological assessments

On every visit, the following neuropsychological tests were administered: MMSE, Alzheimer Disease Assessment Scale - cognitive subscale (ADAScog) [13], Wechsler Memory Scale - Revised Logical Memory I and II, Auditory Verbal Learning Test, Boston Naming Test, Trail Making Test A and B, Digit Symbol Test (forward and backward), Clock Drawing Test and Category Fluency (animals and vegetables) [14]. For what we term univariate (endpoint-related) analysis, we used the ADAScog modified total score - that is, the traditional ADAScog total score plus Delayed Word Recall and Digit Cancellation with a maximum score of 85 (Table 1). For what is termed multivariate (trajectory-related) analysis, we computed a neuropsychological composite score (Neuro-Psychological Battery (NP-Batt)) as defined by Cronk and colleagues [15]. This is the average of nine *z *scores determined by standardization with means and standard deviations (SDs) of the normal controls of the ADNI database. The nine subtests are: Logical Memory II, Digit Span Forward, Digit Span Backward, category fluency animals, category fluency vegetables, Trail Making B, Boston Naming Test, Auditory Verbal Learning Test, and Digit Symbol Test.

Response variables for our models were ADAScog at 24 months and NP-Batt scores at all follow-up visits. We used baseline evaluations of these scores, plus demographics (age, gender, years of education, body mass index, number of apolipoprotein E4 alleles), the Hachinski modified score, the total score of the FAQ [16] and the MMSE score as potential predictors in our models. The body mass index is divided into three classes: <25 kg/m^2^, ≥25 to <30 kg/m^2^, ≥30 kg/m^2^. In supplementary analyses with a smaller subject sample we also included the ratio of the cerebrospinal fluid biomarkers β-amyloid_1-42 _(Aβ42) over total tau protein (T-tau) as a potential predictor.

### Statistical analysis

In the univariate analysis we forecast the ADAScog after 24 months from demographic variables, apolipoprotein E4, body mass index, modified Hachinski score, and baseline values of FAQ, MMSE, ADAScog and NP-Batt scores in a linear regression model. Twenty-four months were chosen because this is a time span in which significant cognitive decline may be expected in MCI subjects and because there were many missing values in the database at later times. As the distribution of ADAScog scores is heavily skewed to the right, a square-root transformation was applied. Variable selection is based on the Akaike Information Criterion [17] and, after the main effects model is found, all pairwise interactions are tested for inclusion in this model. Simulated control group data are generated by randomly generating a parameter vector from the multivariate normal distribution of the estimated parameter vector, by applying this parameter vector to the covariate values of the individual considered (that is, forming the linear combination), and by adding random noise with the variance of the residuals. Power calculation is based on the assumption that treated individuals would have a lower ADAScog score at 24 months (that is, less deterioration) than untreated controls. The standard power formula for comparison of two means is applied. Since a randomized treatment is uncorrelated with other covariates, this formula drops from the more general procedure for parameters in regression models.

The multivariate model is a mixed model with random intercepts and random slopes versus time [18]. It is computed for the NP-Batt score at all follow-up visits. We include the same predictors as those in the univariate analysis as well as the interactions of these predictors with time in a starting model, and eliminate effects in a stepwise manner based on their *P *values. We then test whether pairwise interactions of main effects that stayed in the model should be included. Time is coded as the visit number in units of 6 months; that is, the visit at month 36 has the visit number 6. All slopes on time are changes per visit. Simulated control group data are generated in a similar manner as in the univariate model with additional simulation of individual random effects. The power is determined by simulation: we simulated 1,000 treatment datasets (with the covariates of the ADNI MCI) with slope -0.74 and compared each of them with the given control group, which had a slope of -0.94.

For both types of models we display effects in profile plots. We compare the simulated response variables with their observed counterparts. The validity of the model was checked by fivefold cross-validation; that is, the dataset was randomly split into five equally sized subsets, responses of each subset were predicted by a model estimated from the union of the other subsets and the overall prediction error was determined by comparing all predictions with the actual observed responses. As co-linearity might be a concern, we computed the variance inflation factor of each quantitative predictor: they were all below 1.70 (that is, a fairly low value).

## Results

Preliminary analysis with the number of apolipoprotein E4 alleles placed into three categories (0, 1, 2) showed that two alleles approximately duplicated the effect of one allele. We therefore put the number of E4 alleles as a numerical predictor in our models.

### Univariate analysis

Starting with all potential predictors of the square-root-transformed ADAScog total at 24 months (see Baseline data and neuropsychological assessments), years of education, age, Hachinski score and the number of E4 alleles were eliminated in backward stepping with the Akaike Information Criterion. Subsequent testing for single pairwise interactions yielded four significant results, but only one - the interaction of FAQ with NP-Batt - remained in the model after another Akaike Information Criterion-based elimination. The ADAScog total at baseline seems to be the strongest predictor in this model (Table 2).

**Table 2:** Results of the univariate regression model for the ADAScog total at month 24

	Estimate	Standard error	*t *value	*P *value
Intercept	3.84232	0.76473	5.02	<0.0001
Gender	0.28474	0.08387	3.39	0.0008
Obesity	-0.27526	0.11680	-2.36	0.019
FAQ at baseline	0.07902	0.01787	4.42	<0.0001
MMSE at baseline	-0.06994	0.02534	-2.76	0.0062
ADAScog at baseline	0.09250	0.00768	12.04	< 0.0001
NP-Batt at baseline	-0.50529	0.08820	-5.73	<0.0001
FAQ × NP-Batt at baseline	0.03762	0.01341	2.81	0.0054

*R*^2 ^= 0.6344, standard deviation (residuals) = 0.6692. ADAScog, Alzheimer Disease Assessment Scale - cognitive subscale; FAQ = Functional Assessment Questionnaire; MMSE, Mini-Mental State Examination; NP-Batt, Neuro-Psychological Battery.

Fivefold cross-validation with 20% of the responses predicted in each run achieved a mean prediction error of 0.671, very close to the residual SD of 0.669. This confirms the stability of the model.

**Figure 1:**
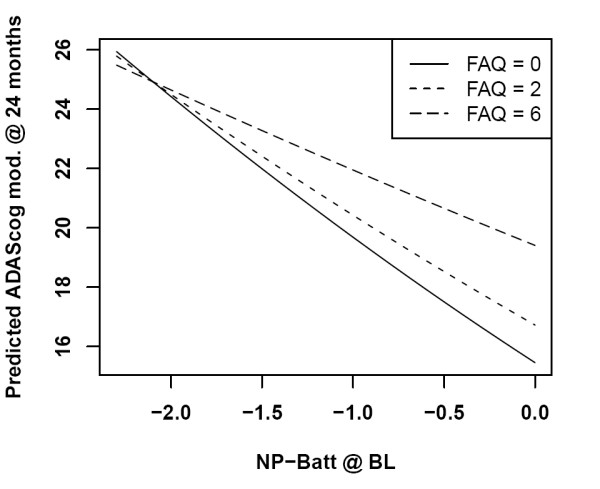
**Interaction of the Functional Assessment Questionnaire with the Neuro-Psychological Battery**. Interaction of the Functional Assessment Questionnaire (FAQ) with the Neuro-Psychological Battery (NP-Batt) in a univariate model for the Alzheimer Disease Assessment Scale - cognitive subscale (ADAScog) total score. Lines are shown for quartiles of the FAQ. BL, baseline; mod., modified.

The interaction of FAQ with NP-Batt (both at baseline) is displayed as a profile plot with FAQ fixed at its quartiles (Figure 1). At low values of the NP-Batt score, the ADAScog total is not dependent on the FAQ; but at normal values of the NP-Batt, the ADAScog total score is some 5 units higher if the FAQ score is 6 instead of 0 (*P *= 0.005 for the interaction).

**Figure 2:**
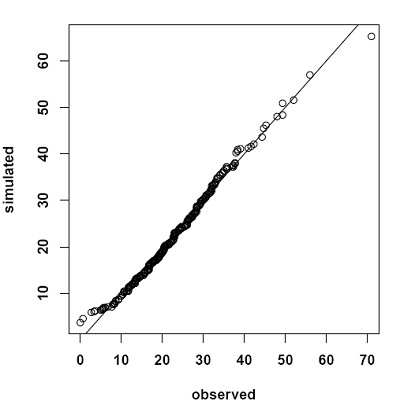
**Simulated versus observed Alzheimer Disease Assessment Scale - cognitive subscale data**. Quantile-quantile plot of simulated versus observed Alzheimer Disease Assessment Scale - cognitive subscale total scores at month 24.

Based on the regression model shown in Table 2 we simulated ADAScog scores after 24 months. The simulated sample (mean 22.1, SD 10.5) showed a very similar distribution to the observed sample after 24 months (mean 22.4, SD 10.0). Figure 2 shows a quantile-quantile plot of simulated versus observed data.

An effective AD treatment is expected to attenuate the increase of the ADAScog total score at month 24 in treated compared with untreated MCI subjects. We assume an effect of 0.16 on the square-root scale of ADAScog, corresponding to an improvement of 1 point at an ADAScog score of 10, or of 2.5 points at an ADAScog score of 60. The analytically derived power with sample sizes of 286 per group and based on the estimated SD of the residuals (0.669) in each group is 82%.

### Multivariate analysis

The multivariate model is set up with the NP-Batt score as the outcome. Note that higher NP-Batt scores indicate better performance. Model selection follows the proposal of Verbeke and Molenberghs [18]. The starting model is saturated with all fixed main effects, with all fixed interactions with time and with random intercepts and random slopes. This model is significantly better (*P *< 0.0001, likelihood ratio test) than the model that drops random slopes. The model hence allows for individual regression lines with varying intercepts and slopes for each patient. Backward stepwise elimination of the fixed interactions with time and the fixed main effects, as well as inclusion of other pairwise interactions, is based on Wald tests. The resulting final model is displayed in Tables 3 and 4. NP-Batt at baseline is the strongest main effect (*t *= 32.3, *P *< 0.0001) with a coefficient of 0.97, which is close to 1.

**Table 3:** Fixed effects of the mixed model for the Neuro-Psychological Battery

Fixed effects	Value	Standard error	Degrees of freedom	*t *value	*P *value
Intercept	0.92879	0.22683	1,065	4.095	< 0.0001
BMI-2 (category 25 to 30 kg/m^2^)	-0.06911	0.03812	366	-1.813	0.0706
BMI-3 (category >30 kg/m^2^)	-0.04498	0.05337	366	-0.843	0.3999
Visit number	0.01241	0.02184	1,065	0.568	0.5701
Apolipoprotein E4	0.03383	0.02608	366	1.298	0.1953
Age	-0.00887	0.00292	366	-3.040	0.0025
FAQ at baseline	-0.11273	0.03835	366	-2.939	0.0035
ADAScog at baseline	-0.00299	0.00339	366	-0.882	0.3782
NP-Batt at baseline	0.97529	0.03019	366	32.303	<0.0001
(BMI-2) × Visit number	0.02130	0.01366	1,065	1.559	0.1192
(BMI-3) × Visit number	0.05513	0.02003	1,065	2.752	0.0060
Visit number × apolipoprotein E	-0.03558	0.00932	1,065	-3.818	0.0001
Visit number × ADAScog	-0.00557	0.00104	1,065	-5.374	<0.0001
Age × FAQ	0.00140	0.00051	366	2.723	0.0068

Standard deviation (residuals) = 0.2175. ADAScog, Alzheimer Disease Assessment Scale - cognitive subscale; BMI, body mass index; FAQ, Functional Assessment Questionnaire; NP-Batt, Neuro-Psychological Battery.

**Table 4:** Random effects of the mixed model for the Neuro-Psychological Battery

Random effects	Lower limit	Value	Upper limit
SD(intercept)	0.1424	0.1856	0.2419
SD(slope)	0.0537	0.0677	0.0852
Cor(intercept; slope)	-0.2694	0.6544	0.9510
Within-group SD	0.2377	0.2500	0.2629

Standard deviation (SD) and correlation coefficient (Cor) with 95% confidence intervals.

**Figure 3:**
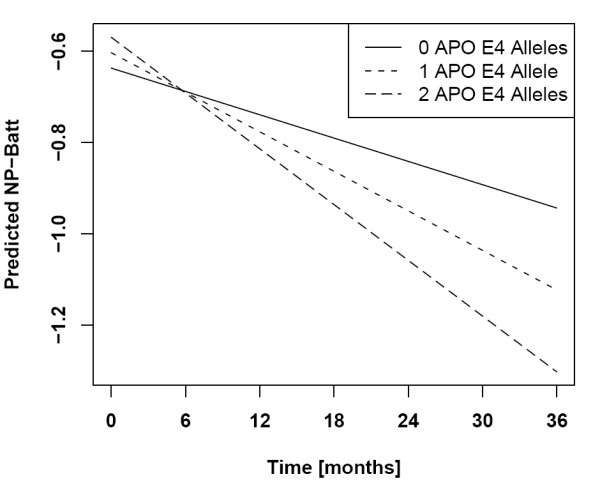
**Interaction of apolipoprotein E4 with the Neuro-Psychological Battery**. Interaction of the number of apolipoprotein E4 (APO E4) alleles with time in the multivariate model for the Neuro-Psychological Battery (NP-Batt).

Obese individuals have a flatter slope of the NP-Batt score over time than normal weighted individuals (interaction *P *= 0.006). Higher ADAScog baseline scores increase the slope (graph not shown; interaction *P *< 0.0001). The number of E4 alleles also increases this slope (interaction *P *= 0.0002). We display the effect of the number of E4 alleles with time in Figure 3, the plot of this interaction showing a moderate decrease of performance in MCI subjects without an E4 allele and a steeper decrease in subjects with one or even two E4 alleles.

Fivefold cross-validation confirmed the stability of this model. The mean prediction error is 0.2176, which is very close to the corresponding quantity computed from the residuals (0.2175). The distribution of simulated NP-Batt values for visits at 6, 12, 18 and 24 months, based on the demographic and baseline values of the 375 MCI patients in the ADNI database, was very similar to the observed data (Table 5).

**Table 5:** Descriptive statistics of observed and simulated NP-Batt scores for MCI at months 6, 12, 18 and 24

	Minimum	First quartile	Median	Mean	Third quartile	Maximum	Standard deviation
Observed	-3.95	-1.50	-0.97	-1.01	-0.47	1.74	0.83
Simulated	-4.12	-1.59	-1.00	-1.04	-0.45	1.85	0.85

MCI, mild cognitive impairment; NP-Batt, Neuro-Psychological Battery.

NP-Batt scores of patients without an E4 allele decreased with an average slope of -0.094. We hypothesize that an effective treatment for secondary prevention of dementia due to AD might improve this slope by 0.02 to an average slope of -0.074 per visit. Simulating treatment data with this (alternative) hypothesis and comparing them with the observed ADNI data as placebo control achieves a power of about 80% (1,000 simulations).

### Supplementary analysis including cerebrospinal fluid biomarkers

**Figure 4:**
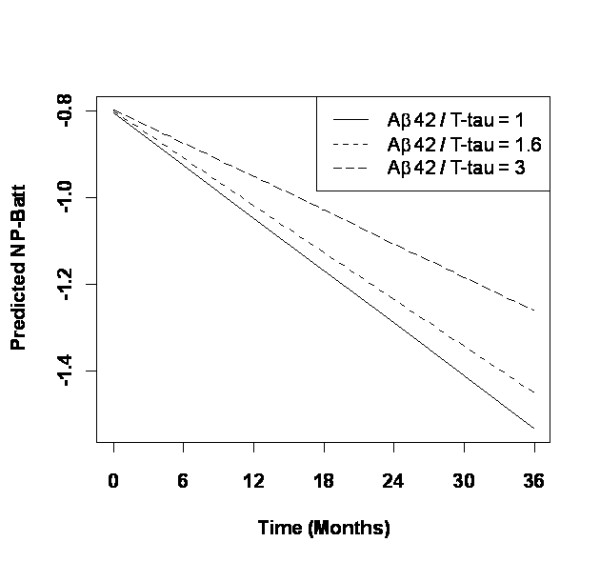
**Interaction of amyloid β42/total tau protein with time**. Interaction of amyloid β42 (Aβ42)/total tau protein (T-tau) with time in the multivariate model for the Neuro-Psychological Battery (NP-Batt) (189 patients).

The same model selection strategy as above is used for the analysis of NP-Batt with the additional covariate Aβ42/T-tau in the cerebrospinal fluid [19]. Random slopes are again included in the model. The resulting final model after elimination of most interactions and some fixed effects is displayed in Tables 6 and 7. NP-Batt is still the most significant predictor (*t *= 22.9, *P *< 0.0001) with a regression coefficient of 1.00. Higher values of Aβ42/T-tau lead to flatter slopes of NP-Batt scores over time. This interaction is displayed in Figure 4 for Aβ42/T-tau values of 1, 1.6 and 3; that is, close to the quartiles and median value of this covariate. The interaction of time with ADAScog baseline scores is somewhat stronger than in the previous analysis (*P *= 0.0001).

**Table 6:** Fixed effects of covariates including Aβ42/T-tau in the mixed model for NP-Batt (189 patients)

Fixed effects	Value	Standard error	Degrees of freedom	*t *value	*P *value
Intercept	0.19832	0.09375	564	2.115	0.0348
Visit number	-0.07163	0.03346	564	-2.141	0.0327
Aβ42/T-tau	0.00328	0.01535	184	0.213	0.8312
FAQ at baseline	-0.01015	0.00508	184	-1.999	0.0472
ADAScog at baseline	0.00058	0.00463	184	0.125	0.9007
NP-Batt at baseline	0.99973	0.04373	184	22.861	<0.0001
Visit number × (Aβ42/T-tau)	0.02229	0.00548	564	4.065	0.0001
Visit number × ADAScog	-0.00398	0.00146	564	-2.719	0.0067

Aβ42, β-amyloid_1-42_; ADAScog, Alzheimer Disease Assessment Scale - cognitive subscale; FAQ, Functional Assessment Questionnaire; NP-Batt, Neuro-Psychological Battery; T-tau, total tau protein.

**Table 7:** Random effects of the mixed model for NP-Batt including Aβ42/T-tau as covariate (189 patients)

	Lower limit	Value	Upper limit
SD(intercept)	0.1236	0.1816	0.2669
SD(slope)	0.0506	0.0698	0.0962
Cor(intercept, slope)	-0.5034	0.5598	0.9487
Within-group SD	0.2342	0.2512	0.2693

Standard deviation (SD) and correlation coefficient (Cor) with 95% confidence intervals. Aβ42, β-amyloid_1-42_; NP-Batt, Neuro-Psychological Battery; T-tau, total tau protein.

Fivefold cross-validation again confirmed the stability of the model including Aβ42/T-tau. The mean prediction error coincides with the residual standard deviation - both are 0.2187, close to the prediction error achieved from the full sample.

## Discussion

The main goal of the current study is to develop mathematical models of typical disease trajectories of AD, from its presymptomatic to symptomatic stages - that is, to develop algorithms for use to quantitatively compare patients undergoing experimental treatment for secondary prevention of dementia due to AD with their own anticipated untreated disease course. We used the data for 397 MCI subjects from the ADNI database as available in October 2009. The examples presented here concern a univariate (endpoint-related) approach - that is, an algorithm that predicts the MCI subject group's performance scores on the ADAScog 24 months after their baseline examination - and a multivariate (trajectory-related) approach - that is, an algorithm that forecasts the decline of performance during 36 months, from baseline to the last examination after 3 years - on the composite score of a neuropsychological battery as described previously (NP-Batt) [15]. Both outcomes, a cognitive performance score after 24 months and the trajectory of cognitive change over 36 months, could be of use in studies with experimental drugs for secondary prevention of dementia due to AD. A total of 11 demographic, neuropsychological and biological measures established at baseline, plus their interactions, were included as potential predictors in the univariate and multivariate analyses.

In the univariate model, the strongest predictors of the ADAScog scores as measured after 24 months were the ADAScog scores established at baseline. Other significant predictors were (in decreasing order of importance) the composite scores on the NP-Batt, the MMSE scores, gender and obesity. There was also a significant interaction of the FAQ with the relationship between the NP-Batt at baseline and the ADAScog scores at 24 months, suggesting that higher scores on the FAQ (indicating some functional restriction at baseline) mainly affected 24 months ADAScog scores of MCI subjects with normal NP-Batt scores at baseline, but not those with lower baseline performance.

In the multivariate analysis, the strongest predictors of the NP-Batt trajectory over 36 months were the NP-Batt scores established at baseline. Other significant, albeit weaker, predictors are age, the FAQ scores at baseline and obesity. Of particular interest is the interaction between the number of apolipoprotein E4 alleles and time (Figure 3), indicating that the negative impact of this genetic marker upon cognitive performance was not significant at baseline but developed over time.

Taken one by one, none of these findings is novel. For the baseline cognitive measures (ADAScog, MMSE, NP-Batt) predicting cognitive performance after 24 months as well as the trajectory of cognitive decline over 36 months, numerous studies show that cognitive performance established at some point in time (memory function and other cognitive domains) is a strong predictor of subsequent cognitive decline and of dementia. This was found, at different levels of performance, for older subjects who were cognitively healthy at baseline [20,21], for MCI subjects [22] and for AD patients [6,23]. The concept of cognitive reserve [24,25] captures these observations in a more general hypothetical construct.

The effect of gender was significant in the univariate approach, but not in the multivariate model. While it is known from epidemiological studies that AD occurs more frequently in aged women than in men [26], this general finding does not explain the difference in the two models.

Obesity was found to have a protective effect against cognitive decline in both the univariate model and the multivariate model. Cronk and colleagues, who also worked with the ADNI database, have already reported a favorable impact of higher body mass index baseline values on the development of MMSE, ADAScog and NP-Batt scores as early as 1 year after baseline [15]. Other authors also found a protective effect of obesity upon cognitive performance in persons of older age [27,28]. Although overweight in middle age was identified as a risk factor for dementia several decades later [29,30], this relationship appears to be reversed in persons beyond 70 years of age (the obesity paradox [28]). To what extent this paradox and the apparent protective effect of obesity are due to an underlying selection factor - for example, higher mortality in overweight people lacking a hypothetical protective factor that also supports cognitive maintenance - cannot be deducted from the current data.

There was a significant interaction between the FAQ scores and the NP-Batt scores as predictors of the performance on the ADAScog at 24 months. FAQ scores at baseline were a significant determinant of cognitive decline in the multivariate analysis when NP-Batt scores were still normal (that is, *z *= 0). As mentioned earlier, the ADNI MCI sample cannot be considered a pure selection of MCI subjects since some subjects had restrictions in activities of daily life and instrumental activities of daily life, as indicated by baseline FAQ scores >0 (Table 1), and 60% of the subjects were taking anti-AD drugs (cholinesterase inhibitors and/or memantine) at some point in the study. With regard to impaired activities of daily life/instrumental activities of daily life in some subjects, it is of interest that the baseline FAQ scores turned out to be a significant and, at least in the multivariate model, independent predictor of cognitive decline over the ensuing 36 months. According to Pérès and colleagues, some decline in instrumental activities of daily life is seen in aged subjects as early as 10 years before a clinical diagnosis of dementia is made, and may thus constitute a very early marker of dementia [31]. Dickerson and colleagues found that, in mildly impaired aged individuals who did not meet strict MCI criteria as implemented in clinical trials, the degree of cognitive impairment in daily life and performance on neuropsychological testing impacted the likelihood of an AD diagnosis within 5 years [32].

As for the possible effect of anti-AD medication, separate analyses for MCI subjects with and without use of cholinesterase inhibitors and/or memantine at any time provided very similar predictive models. Although the patients taking any of these drugs at some time showed somewhat inferior cognitive performance at baseline - that is, higher average ADAScog scores and lower scores on the NP-Batt than subjects not taking any of these drugs - the major predictors for the ADAScog scores after 24 months and the trajectory of the NP-Batt over 36 months were the same for both subgroups (data not shown).

While the number of apolipoprotein E4 alleles was not a significant predictor of the ADAScog scores at 24 months in the univariate analysis, this genetic risk factor of AD showed a significant interaction with time and, consequently, a strong impact upon the trajectory of the NP-Batt scores in the longitudinal analysis (Figure 3). Interestingly, the number of apolipoprotein E4 alleles did no longer significantly affect cognitive performance when the Aβ42/T-tau quotient was introduced as a potential predictor; note, however, that the analysis including the cerebrospinal fluid markers was performed with a sample only one-half as large as that for the other calculations. The Aβ42/T-tau quotient is a known early marker of AD [19,33] and was recently reported to be a predictor of functional decline in the ADNI MCI sample [34]. Aβ42 and subsequently tau in the cerebrospinal fluid are considered early markers in the AD pathological cascade [35].

In summary, although none of the individually significant predictors identified in our models was unexpected, the specific and weighted combination of predictors is novel in the models presented: the univariate approach predicted cognitive performance (ADAScog scores) 2 years after baseline, and the multivariate approach forecasted the decline of cognitive performance - as measured by means of the NP-Batt - over 36 months. The univariate model for the square root of ADAScog explains 63% of the variance, and the prediction error of a single outcome in cross-validation is 0.67. This is obviously not a precise estimate for single values, as the 95% interval for an estimate of ADAScog score of 20 would range roughly from 10 to 34 for an individual patient. In the application of this model to clinical studies, however, the relevant measure will be the mean of, say, 200 outcomes. The standard error of this mean would be 0.047 on the square-root scale, and the 95% confidence interval for a mean of 20 on the original scale would be as narrow as 19.2 to 20.8. In the multivariate model for NP-Batt, which has a standard normal distribution in a healthy population, the standard deviation of the conditional residuals is 0.2175. Taking the between-patient variability into account, the standard deviation of residuals in the population is between 0.32 and 0.51, depending on the time of observation (0 to 36 months). For a sample of 200 subjects these standard deviations are reduced to 0.023 and 0.035, respectively. Estimated means from such samples appear to be sufficiently precise for group comparisons in clinical trials.

An important point to be addressed in future analyses concerns the possibility of generalizing our models to new, independent datasets and, eventually, their application in clinical trials of experimental drugs intended for secondary prevention of dementia due to AD. As a first step in this direction, the simulation model for the NP-Batt as derived from the ADNI MCI subjects was challenged by applying it to the AD patient sample of the ADNI dataset. NP-Batt data for visits at 6, 12 and 24 months were simulated (there are no data from AD patients at 18 months). A comparison of the scores from the observed and the simulated data is shown in Table 8 and indicates that the observed and the model-based simulated values of the ADNI AD patient sample were indeed very similar. This is a preliminary indication that the mathematical model established from MCI data is also usable for datasets from patients with dementia; that is, over a wide range of presymptomatic and symptomatic AD patients. We are currently testing our models with MCI datasets from other, ADNI-independent projects, which contained partly different assessment criteria. The results of these tests will be reported in due time.

**Table 8:** Descriptive statistics of observed and simulated NP-Batt scores for Alzheimer's disease patients at months 6, 12 and 24

	Minimum	First quartile	Median	Mean	Third quartile	Maximum	Standard deviation
Observed	-4.48	-2.66	-1.95	-2.05	-1.40	-0.14	0.87
Simulated	-4.85	-2.65	-2.00	-2.08	-1.45	0.10	0.89

Simulation based on the model for mild cognitive impairment individuals. NP-Batt, Neuro-Psychological Battery.

Assuming that our models are supported by analyses of further independent datasets, what would principally argue against their use in trials with experimental compounds aimed at secondary prevention of dementia due to AD? Evidently, the 50-year tradition of placebo-controlled study designs in clinical neuropsychopharmacology argues against a new approach like the one suggested here - although several limitations of the conventional designs have repeatedly been pointed out [4,7,8].

To illustrate one particularly important limitation of RPCTs and its consequences, let us for a moment assume the perspective of an older person who has just learned that he or she shows the characteristics of presymptomatic AD or MCI, implying that he or she is likely to become demented within a few years, and who is offered participation in a long-term phase 3 RPCT with a promising experimental disease-course altering drug. Would one not expect that this individual would be uncertain as to how he or she should decide: agree to participate in a placebo-controlled - that is, a Russian roulette type of trial - or reject participation and hope for a better alternative?

In recent years, clinical investigators - notably in the United States - have reported increasing difficulties recruiting patients into AD clinical trials [36]. One cannot rule out that some of these difficulties are due to patients' unwillingness to enter trials that entail a high risk for participants of being treated with placebo for months or even years. Thus, apart from the ethical issue of exposing high-risk individuals to admittedly ineffective treatment (placebo), one should also consider that only a self-selected fraction of all trial candidates will eventually enter RPCTs, a fact that seriously jeopardizes the external validity of such trials. In spite of these concerns, current regulatory guidelines [37] and specialized task forces [38,39] keep recommending or even demand RPCTs as proof of efficacy for drugs intended for use in AD, including compounds aimed at secondary prevention of dementia due to AD that require very long studies to prove efficacy. This insistence is surprising, given that placebo-controlled designs were originally developed for clinical studies of analgesics, antidepressants and anxiolytics - that is, for trials in mostly self-limiting, unstable and partly subjective central nervous system indications that differ in important aspects from slowly developing, irreversible, degenerative disorders such as AD.

If supported by further evidence, where in the clinical development process of a new compound aimed at secondary prevention of dementia due to AD could be the place for the proposed PGSA? No doubt some of the earlier (phase 1 and phase 2) trials, which are often performed on healthy subjects and subsequently on AD patients at different levels of deterioration, do require placebo control, notably in order to detect and characterize any relevant safety issue of the new compound. As these earlier studies last only a few months for each patient, and since little is known early in development about a new drug's potentially useful effect in man, there is no ethical concern to use placebo at this stage. Once the proof-of-principle and placebo-controlled safety studies are completed, however, and presumably effective and safe doses of the novel drug need to be tested for long-term efficacy in the target population - that is, in subjects in presymptomatic stages of AD - then a placebo-free approach such as the PGSA should be seriously considered. In addition to its ethical and scientific merits, it also has the potential to save patients, time and money. The next years will show whether the AD research community [3,7,40,41] and drug regulatory bodies are ready and willing to de-emphasize a traditional study paradigm that has serious shortcomings, and are willing to consider a design that has the potential both to benefit the patients and facilitate anti-AD drug development.

## Conclusions

First predictive univariate (endpoint-related) and multivariate (trajectory related) models based on anamnestic, clinical, biological and neuropsychological data from the ADNI database show high correspondence of predicted and real observed values. Corroboration of these models with data from other studies is ongoing. It is hoped that the PGSA, which comprises comparisons between real, observed data of patients on experimental treatment with their own, model-based forecasted trajectories, will be considered for late phase 2 or phase 3 long-term trials with drugs intended for secondary prevention of dementia due to AD.

## Competing interests

RS, MB, ARM and AUM have applied for an international patent covering the PGSA. The authors declare they have no other competing interests.

## Abbreviations

Aβ42: amyloid β42; AD: Alzheimer's disease; ADAScog: Alzheimer Disease Assessment Scale - cognitive subscale; ADNI: Alzheimer Disease Neuroimaging Initiative; FAQ: Functional Assessment Questionnaire; MCI: mild cognitive impairment; MMSE: Mini-Mental Status Examination; NP-Batt: Neuro-Psychological Battery; PGSA: placebo group simulation approach; RPCT: randomized placebo-controlled trial; SD: standard deviation; T-tau: total tau protein.

## Authors' contributions

RS is the originator of the principle of the PGSA and wrote major parts of the manuscript. MB developed the mathematical models underlying the PGSA. ARM and AUM made important intellectual contributions to the development of the PGSA and provided relevant input to the manuscript.
